# Prospective association between circadian syndrome and incident chronic lung disease in the CHARLS and ELSA cohorts

**DOI:** 10.1038/s41598-026-50994-1

**Published:** 2026-05-16

**Authors:** Boheng Liu, Shurui Wu, Yan Xu, Jipeng Jiang, Yang Liu

**Affiliations:** 1 Postgraduate School, Medical School of Chinese PLA, Haidian, Beijing People’s Republic of China; 2 Department of Thoracic Surgery, The First Medical Center of Chinese PLA General Hospital, Haidian, Beijing People’s Republic of China

**Keywords:** Circadian syndrome, Chronic lung disease, Chronomedicine, Epidemiology, Cohort study, Sleep quality, Biomarkers, Diseases, Endocrinology, Health care, Medical research, Risk factors

## Abstract

Circadian syndrome (CircS) involves the integration of systemic circadian disruption with cardiometabolic risk factors, but its longitudinal effect on incident chronic lung disease (CLD) remains unknown. This prospective dual-cohort study analyzed data from the China Health and Retirement Longitudinal Study (CHARLS, *n* = 7,553) and the English Longitudinal Study of Ageing (ELSA, *n* = 4,957). CircS was defined by the clustering of at least 4 out of 7 circadian-metabolic components. Multivariate Cox proportional hazards models estimated hazard ratios (HRs) for incident CLD over a seven-year follow-up. In the discovery cohort (CHARLS), CircS was independently associated with an increased risk of incident CLD (*HR* = 1.16, 95% *CI* = 1.01–1.33). This was robustly confirmed in the validation cohort (ELSA) with a stronger effect size (*HR* = 1.53, 95% *CI* = 1.20–1.95). Furthermore, subgroup analyses revealed that this risk was significantly amplified by alcohol consumption in the British population (*P* < 0.05), suggesting an interactive effect between circadian misalignment and lifestyle stressors. In conclusion, CircS serves as a robust, independent predictor of respiratory decline. Evaluating circadian integrity offers a reliable indicator of respiratory vulnerability, and holistic chronomedicine approaches targeting sleep hygiene and moderate alcohol consumption may help mitigate the global CLD burden.

**Supplementary Information:**

The online version contains supplementary material available at 10.1038/s41598-026-50994-1.

## Introduction

 Chronic lung diseases (CLDs) are leading causes of morbidity and mortality worldwide, and traditional preventive strategies that focus solely on the cessation of smoking and the avoidance of pollutants cannot further decrease morbidity and mortality^1^. New physiological evidence suggests that pulmonary homeostasis is not autonomous but is instead orchestrated by the central circadian clock. The molecular clock regulates important lung defense mechanisms, including airway inflammation, redox balance via the Nrf2 pathway, and mucus secretion^2–4^. Consequently, circadian syndrome (CircS) has emerged as a novel clinical issue and represents the pathological convergence of systemic circadian disruption and metabolic stress^5^. Unlike isolated risk factors, CircS captures the cumulative burden of disturbances in the sleep–wake cycle and cardiometabolic dysregulation, offering a holistic metric of physiological desynchrony^6^.

Although researchers have established a link between circadian clock-associated genes and lung immunity in experimental models, the epidemiological translation of these findings is incomplete and fragmented^7^. Previous studies have adopted primarily an organ-centric perspective, focusing on single variables such as sleep duration or shift work while ignoring the synergistic effect of clustered metabolic and rhythm disturbances^8^. Whether CircS, as a composite phenotype, serves as an independent predictor of respiratory decline remains unknown. Given the distinct environmental and lifestyle patterns in East China and West China, whether the effect of CircS on lung health is a universal biological phenomenon or a context-dependent risk is not clear^9^.

To fill this knowledge gap, we conducted a prospective dual-cohort study using data from the China Health and Retirement Longitudinal Study (CHARLS) and the English Longitudinal Study of Ageing (ELSA). By adopting this study design, we could perform rigorous cross-cultural validation. We aimed to (1) characterize the longitudinal trajectory of the risk of CLD associated with baseline CircS; (2) separate the contribution of CircS from that of traditional lifestyle confounders; and (3) determine whether this association is modified by environmental stressors, such as obesity and alcohol consumption, to identify vulnerable subpopulations.

## Methods

### Study population

To conduct this prospective dual-cohort study, we used data from CHARLS^10^ as the discovery cohort and that from ELSA^11^ as the validation cohort. Both surveys are harmonized longitudinal studies of adults who are at least 45 years old and are designed to represent the Chinese and English populations, respectively. In CHARLS, the baseline data were collected in 2011 (Wave 1), with follow-up extending to 2018 (Wave 4). In ELSA, Wave 6 (2012) served as the baseline data, with outcomes assessed through Wave 9 (2019). Participants were excluded if they met any of the following criteria: (1) were < 45 years old; (2) had a self-reported diagnosis of a CLD (chronic bronchitis or emphysema) or asthma at baseline; or (3) had missing data on key components or covariates of CircS. The final sample comprised 7,553 participants in CHARLS and 4,957 participants in ELSA.

### Assessment of CircS

Circadian syndrome (CircS) was operationalized as a composite phenotype reflecting systemic circadian misalignment^5,12^. As reported in other studies, we defined CircS based on the presence of at least four of the following seven components^13^: (1) Elevated waist circumference: waist circumference cut-offs in CHARLS were ≥ 90 cm for men and ≥ 85 cm for women and these cut-offs in ELSA were ≥ 102 cm for men and ≥ 88 cm for women; (2) High blood pressure: Systolic blood pressure ≥ 130 mmHg, diastolic blood pressure ≥ 85 mmHg, or current use of antihypertensive medication; (3) High triglycerides: Fasting triglyceride levels ≥ 150 mg/dL or specific lipid-lowering treatment; (4) Low HDL-C: High-density lipoprotein cholesterol levels < 40 mg/dL for men or < 50 mg/dL for women, or specific lipid-lowering treatment; (5) High fasting glucose: Fasting glucose levels ≥ 100 mg/dL, HbA1c levels ≥ 5.7%, or self-reported diabetes diagnosis; (6) Short sleep duration: Self-reported sleep duration < 6 h/day; (7) Depression: High depressive symptoms defined by validated cohort-specific cut-offs (≥ 10 on the CES-D 10 for CHARLS; ≥ 4 on the CES-D 8 for ELSA)^14^.

### Determination of incident CLD

The primary outcome was incident CLD, which was defined as a self-reported physician diagnosis of chronic bronchitis or emphysema during the follow-up period. Participants who reported CLD or asthma at baseline were excluded. Incident asthma was explicitly excluded from the primary outcome definition given its distinct etiology, earlier onset, and differing pathophysiology compared with those of chronic bronchitis and emphysema.

The baseline (time origin) for the survival analysis was defined as Wave 1 (2011) for CHARLS and Wave 6 (2012) for ELSA. Only participants without CLD or asthma at baseline were included in the risk set. Incident CLD was established on the basis of the first newly reported physician diagnosis during the follow-up waves. Specifically, this was determined by an affirmative response to the questionnaire item: “Have you been diagnosed with chronic lung diseases, such as chronic bronchitis or emphysema, by a doctor?’ in CHARLS, and ‘Has a doctor ever told you that you have chronic lung disease such as chronic bronchitis or emphysema?’ in ELSA. Because exact clinical diagnosis dates were unavailable, the time-to-event for incident cases was approximated as the midpoint between the interview date when the disease was first reported and the date of the previous interview. Participants who did not develop the outcome were right-censored at one of the following events, whichever occurred first: (1) the date of documented death (ascertained via exit or end-of-life interviews); (2) the date of their last available interview for those lost to follow-up; or (3) the administrative end of the follow-up period (Wave 4 [2018] for CHARLS and Wave 9 [2019] for ELSA). Individuals completely lacking follow-up data were excluded from the longitudinal analysis^15^.

### Covariates and data harmonization

Covariates were carefully selected on the basis of established epidemiological risk factors for respiratory disease. To ensure rigorous comparability between the cohorts, data harmonization was conducted following a validated protocol established in other studies. Sociodemographic covariates included age, sex, and marital status^12^. The level of education was dichotomized into two categories: elementary school or below and middle school or above^12^. With respect to lifestyle behaviors, individuals were classified on the basis of smoking status as a smoker or a nonsmoker, while individuals were classified on the basis of alcohol consumption status as a drinker or a nondrinker. Body mass index (BMI) was derived from height and weight measurements and was treated as a continuous variable or categorized on the basis of population-specific criteria^16,17^. Crucially, to prevent overadjustment bias, the constituent components of CircS, including blood pressure, fasting glucose levels, and depression scores, were intentionally excluded from the covariate adjustment models.

### Statistical analysis

The baseline characteristics of participants with and without CircS were compared by Student’s t tests for continuous variables and chi-square tests for categorical variables. To evaluate the longitudinal association between CircS and incident CLD, we constructed three sequential Cox proportional hazards regression models as follows: (1) Model 1 (unadjusted) assessed the crude association; (2) Model 2 (demographic-adjusted) adjusted for age, sex, marital status, and level of education; and (3) Model 3 (fully adjusted) further adjusted for lifestyle factors (smoking status and alcohol consumption) to control for behavioral confounders.

The proportional hazards assumption was verified using Schoenfeld residuals. No material violation of the proportional hazards assumption was observed for the primary exposure (CircS) in the fully adjusted models for either the CHARLS (*χ*^*2*^ = 0.255, *P* = 0.613) or ELSA (*χ*^*2*^ = 0.050, *P* = 0.822) cohorts. The global test statistics were also nonsignificant (CHARLS: global *χ*^*2*^ = 12.647, *P* = 0.081; ELSA: global *χ*^*2*^ = 7.586, *P* = 0.475). For subgroup analyses, the data were stratified by sex, smoking status, alcohol consumption, and obesity; statistical interactions were tested by entering a product term into the fully adjusted models^18^. To rigorously assess the robustness of our findings against potential bias from missing data, we performed multiple imputation by chained equations (MICE)^19^. Five imputed datasets were generated for each cohort, and the data were assumed to be missing at random (MAR). Cox regression estimates were pooled according to Rubin’s rules. All the statistical analyses were performed using Stata (Version 17.0) and R (Version 4.2.0). All the differences were considered statistically significant at *P* < 0.05 (two-tailed).

Finally, to further account for baseline confounding and ensure the optimal comparability of the binary exposure groups, we applied inverse probability of treatment weighting (IPTW) as a robust sensitivity analysis. Propensity scores were estimated using a multivariable logistic regression model incorporating baseline covariates (age, sex, annual household expenditure, marital status, education level, smoking status, and alcohol consumption). Stabilized weights were calculated to improve baseline comparability between participants with and without CircS. To minimize the influence of extreme weights, these stabilized weights were truncated at the 1st and 99th percentiles. Weighted Cox proportional hazards models incorporating robust standard errors were then fitted to calculate the HRs and 95% CIs.

### Ethical approval and informed consent

The current study is a secondary analysis of publicly available, de-identified datasets from the CHARLS and ELSA cohorts. The original CHARLS study was approved by the Biomedical Ethics Review Committee of Peking University (approval number: IRB00001052-11015). The ELSA study was approved by the London Multicentre Research Ethics Committee (approval number: MREC/01/2/91). Written informed consent was obtained from all participants before their enrollment in the original surveys. As this study utilized completely anonymized and publicly available data, the requirement for additional ethical approval for this secondary analysis was waived by the institutional review board of the Medical School of Chinese PLA. Furthermore, we confirm that all methods in this study were performed in accordance with the relevant guidelines and regulations, including the Declaration of Helsinki.

## Results

### Study population and participant selection

**Fig. 1:**
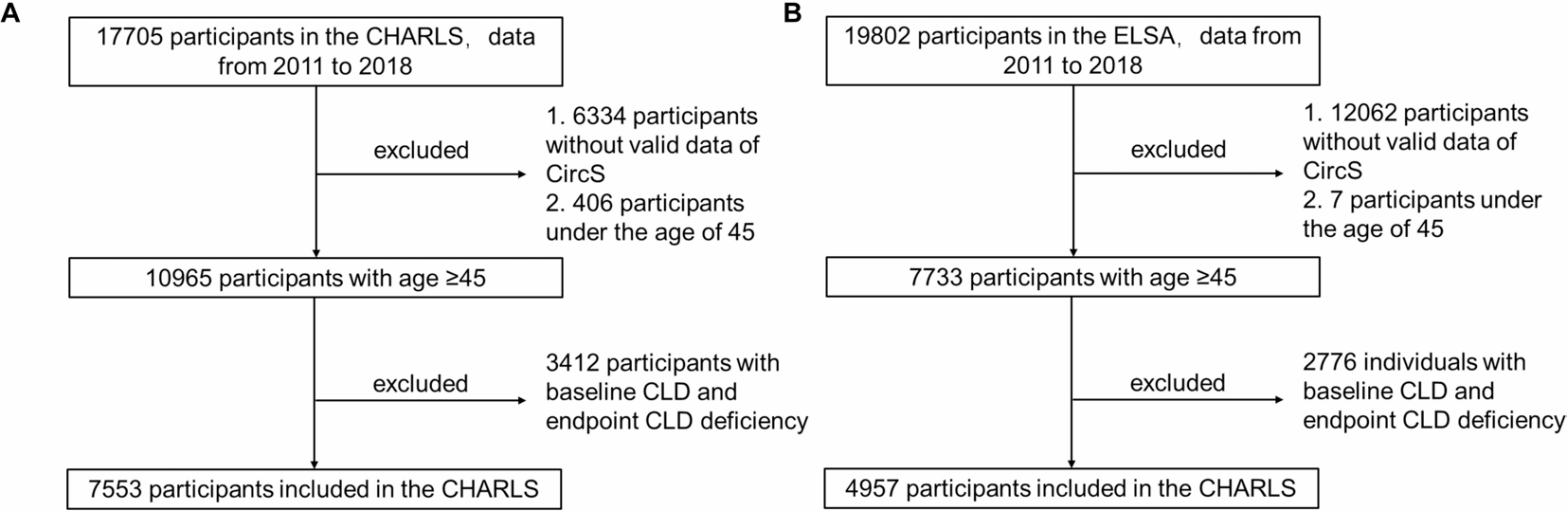
Selection process of the study population in the discovery and validation cohorts. CHARLS, China Health and Retirement Longitudinal Study; ELSA, English Longitudinal Study of Ageing; CircS, circadian syndrome; CLD, chronic lung disease.

The selection process for the study population is illustrated in Fig. 1. In the discovery cohort (CHARLS), 17,705 participants were initially enrolled at baseline (Wave 1). On the basis of the exclusion criteria, we excluded individuals who were < 45 years old, those with missing data on CircS components, and those with invalid baseline or follow-up data on CLD. A total of 7,553 eligible participants were retained for the final analysis. In the validation cohort (ELSA), from an initial pool of 19,802 participants identified during Wave 6, 14,845 individuals were excluded because their data were incomplete or the inclusion criteria were not met. Finally, 4,957 participants were included to validate the associations. The detailed exclusion pathways and sample sizes for both cohorts are presented in Fig. 1A and B, respectively.

### Baseline characteristics and phenotypic heterogeneity

In the discovery cohort (CHARLS), 7,553 participants were included in the final analysis, among which the prevalence of CircS was 38.5%. Individuals with CircS were significantly older (*P* < 0.001), predominantly female, and had a lower level of education than those without CircS did (Table 1).

**Table 1:** Baseline characteristics of the discovery cohort (CHARLS) stratified by CircS status.

	Level	Overall	No	Yes	*p*
*n*		7553	4646	2907	
Age, years (mean (SD))		58.26 (8.79)	57.76 (8.75)	59.07 (8.79)	< 0.001
Sex (%)	Female	4173 (55.2)	2476 (53.3)	1697 (58.4)	< 0.001
	Male	3380 (44.8)	2170 (46.7)	1210 (41.6)	
Marital status (%)	Married and living with spouse	6465 (85.6)	3998 (86.1)	2467 (84.9)	0.004
	Married but not living with spouse	288 (3.8)	193 (4.2)	95 (3.3)	
	Single, divorced, or widowed	800 (10.6)	455 (9.8)	345 (11.9)	
Education (%)	Elementary school or below	5258 (69.6)	3177 (68.4)	2081 (71.6)	0.003
	Middle school or above	2295 (30.4)	1469 (31.6)	826 (28.4)	
Smoking status (%)	Nonsmoker	4712 (62.4)	2815 (60.6)	1897 (65.3)	< 0.001
	Smoker	2840 (37.6)	1830 (39.4)	1010 (34.7)	
Drinking status (%)	Drinker	2085 (29.0)	1337 (30.5)	748 (26.7)	0.001
	Nondrinker	5095 (71.0)	3046 (69.5)	2049 (73.3)	
Annual household expenditure, RMB (mean (SD))		6852.63 (8636.27)	6845.56 (9114.78)	6863.82 (7819.39)	0.934
Presence of chronic conditions (%)	Yes	1962 (26.0)	1023 (22.0)	939 (32.3)	< 0.001
	No	3126 (41.4)	2075 (44.7)	1051 (36.2)	
	Yes	2465 (32.6)	1548 (33.3)	917 (31.5)	
Incident CLD (%)	No	6306 (83.5)	3924 (84.5)	2382 (81.9)	0.005
	Yes	1247 (16.5)	722 (15.5)	525 (18.1)	
Follow-up time, years (mean (SD))		6.76 (0.91)	6.77 (0.90)	6.74 (0.94)	0.271

In contrast, the prevalence of CircS in the validation cohort (ELSA, *n* = 4,957) was considerably lower (17.8%). Unlike the pattern observed in the Chinese population, no significant differences in age or sex distribution were found between groups (*P* > 0.05). However, the CircS phenotype in the British population was strongly driven by metabolic factors, characterized by a substantially higher BMI and a clustering of social isolation (Table 2).

**Table 2:** Baseline characteristics of the validation cohort (ELSA) stratified by CircS status.

	Level	Overall	No	Yes	*p*
*n* (%)		4957	4076	881	
Age, years (mean (SD))		65.84 (8.42)	65.75 (8.38)	66.24 (8.59)	0.12
Sex (%)	Female	2764 (55.8)	2273 (55.8)	491 (55.7)	1
	Male	2193 (44.2)	1803 (44.2)	390 (44.3)	
Marital status (%)	Married	3419 (71.6)	2885 (73.6)	534 (62.5)	< 0.001
	Single	1353 (28.4)	1033 (26.4)	320 (37.5)	
Education (%)	Elementary school or below	1274 (27.8)	944 (25.0)	330 (40.8)	< 0.001
	Middle school or above	3304 (72.2)	2826 (75.0)	478 (59.2)	
Smoking status (%)	Smoker	2952 (59.6)	2365 (58.0)	587 (66.6)	< 0.001
	Nonsmoker	2005 (40.4)	1711 (42.0)	294 (33.4)	
Drinking status (%)	Drinker	4128 (89.5)	3470 (90.9)	658 (83.1)	< 0.001
	Nondrinker	482 (10.5)	348 (9.1)	134 (16.9)	
Annual household expenditure, GBP (mean (SD))		6438.12 (35747.92)	6754.49 (34340.86)	4981.23 (41609.48)	0.185
Presence of chronic conditions (%)	No	1204 (24.3)	1077 (26.4)	127 (14.4)	< 0.001
	Yes	3753 (75.7)	2999 (73.6)	754 (85.6)	
Incident CLD (%)	No	4793 (96.7)	3957 (97.1)	836 (94.9)	0.001
	Yes	164 (3.3)	119 (2.9)	45 (5.1)	
Follow-up time, years (mean (SD))		7.92 (0.57)	7.93 (0.53)	7.86 (0.73)	0.002

### Incidence and survival analyses

**Fig. 2:**
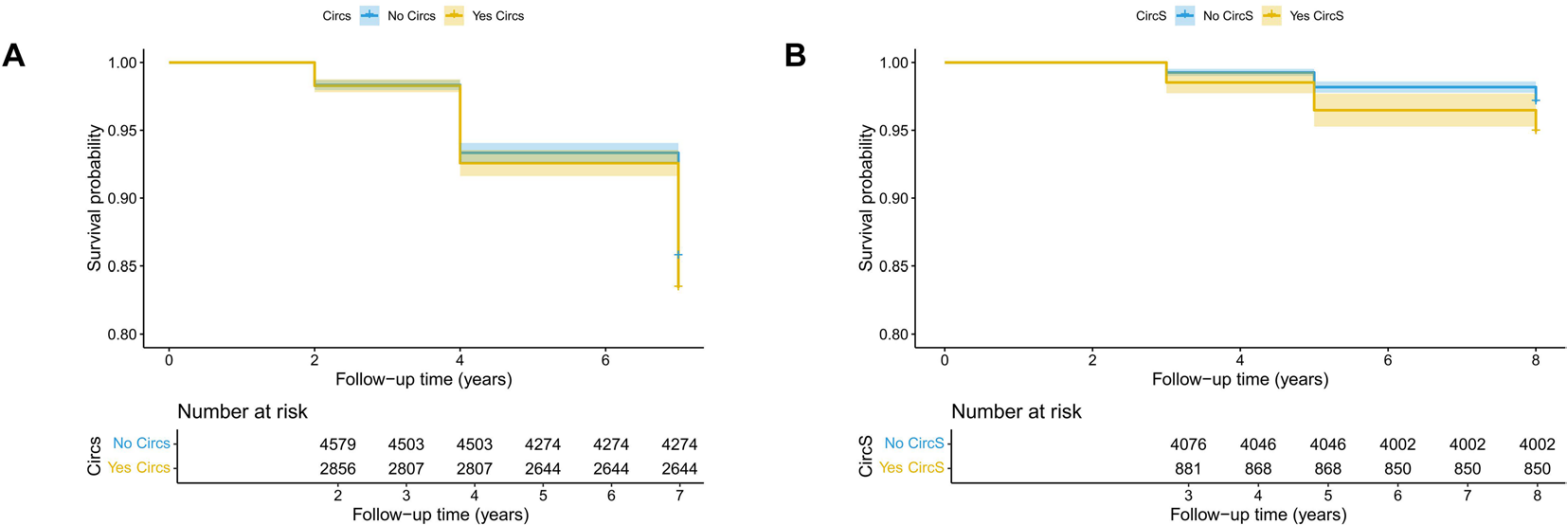
Kaplan–Meier cumulative estimates of CLD incidence stratified by CircS status. (**A**) Discovery cohort (CHARLS) and (**B**) validation cohort (ELSA).

During the seven-year follow-up period, 1,247 and 164 incident CLD cases were identified in the CHARLS and ELSA cohorts, respectively. The results of the Kaplan‒Meier survival analysis demonstrated a prominent difference in respiratory trajectories. In the discovery and validation cohorts, participants with CircS had a significantly higher cumulative incidence of CLD than those without CircS did (log-rank test *P* < 0.001; Fig. 2).

### Association between CircS and incident CLD

We quantified this risk using Cox proportional hazards models. In the discovery cohort (Table 3), CircS was significantly associated with an increased risk of incident CLD. The unadjusted hazard ratio (HR) was 1.173 (95% *CI*: 1.042–1.320). In the fully adjusted model, in which sociodemographic and lifestyle factors were adjusted for (Model 3), CircS was an independent predictor of respiratory decline (*HR* = 1.155; 95% *CI*: 1.005–1.327; *P* = 0.042).

In the validation cohort (Table 3), the effect of CircS was even greater. Although the effect sizes were attenuated after adjustment, the association remained robust. In the fully adjusted model, CircS was associated with a 52.6% higher risk of incident CLD (*HR* = 1.526; 95% *CI*: 1.197–1.946; *P* < 0.001).

**Table 3:** Cox proportional hazards regression analysis of the association between CircS and incident CLD in the CHARLS and ELSA cohorts.

	CHARLS		ELSA	
	HR (95% CI)	*P*	HR (95% CI)	*P*
Model 1	1.173 (1.042–1.320)	0.008	1.677 (1.351–2.082)	< 0.001
Model 2	1.172 (1.033–1.331)	0.014	1.569 (1.247–1.975)	< 0.001
Model 3	1.155 (1.005–1.327)	0.042	1.526 (1.197–1.946)	< 0.001

Model 1: Unadjusted. Model 2: Adjusted for sociodemographic factors (age, sex, marital status, and education level). Model 3: Further adjusted for lifestyle factors (smoking status and alcohol consumption).

### Subgroup analyses

**Fig. 3:**
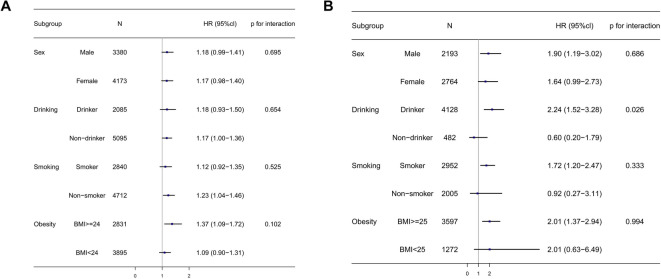
Forest plots of the subgroup analyses for the association between CircS and incident CLD. Hazard ratios (HRs) were estimated using fully adjusted Cox proportional hazards models stratified by sex, smoking status, alcohol consumption, and obesity. (**A**) Discovery cohort (CHARLS) and (**B**) validation cohort (ELSA).

To investigate potential effect modifiers, we performed stratified analyses based on sex, smoking status, alcohol consumption, and obesity (Fig. 3). In CHARLS, the positive association between CircS and the risk of CLD was highly consistent across all the subgroups, with no significant multiplicative interactions observed (*P* > 0.05).

In ELSA, while the direction of the association was mostly consistent, alcohol consumption showed a significant association (Fig. 3). The risk of CLD associated with CircS was greater among current individuals who drank alcohol, whereas the association was not significant among individuals who did not drink alcohol.

### Sensitivity analyses

To assess whether missing covariate data introduced bias into our results, we conducted sensitivity analyses using MICE. The results reinforced the primary findings (Table 4). In the CHARLS cohort, the fully adjusted HR derived from the imputed dataset was 1.213 (95% *CI*: 1.080–1.363; *P* = 0.001). Similarly, in the ELSA cohort, the association remained robust after imputation (*HR* = 1.627; 95% *CI*: 1.108–2.388; *P* = 0.013). Furthermore, in the sensitivity analyses utilizing IPTW to optimize baseline comparability between the CircS and non-CircS groups, the positive associations remained statistically significant and materially unchanged in both cohorts (CHARLS: *HR* = 1.18, 95% *CI*: 1.04–1.35, *P* = 0.011; ELSA: *HR* = 1.59, 95% *CI*: 1.24–2.05, *P* < 0.001) (Supplementary Table S1).

**Table 4:** Sensitivity analysis of the association between CircS and incident CLD conducted using multiple imputation.

	CHARLS		ELSA	
	HR (95% CI)	*P*	HR (95% CI)	*P*
Model 1	1.176 (1.051–1.316)	0.005	1.772 (1.258–2.497)	0.001
Model 2	1.211 (1.079–1.360)	0.001	1.695 (1.157–2.484)	0.007
Model 3	1.213 (1.080–1.363)	0.001	1.627 (1.108–2.388)	0.013

Model 1: Unadjusted. Model 2: Adjusted for sociodemographic factors (age, sex, marital status, and education level). Model 3: Further adjusted for lifestyle factors (smoking status and alcohol consumption).

## Discussion

This study is the first prospective study to characterize the longitudinal trajectory of the risk of CLD associated with CircS across distinct genetic and cultural contexts. Using harmonized data from the CHARLS and ELSA cohorts^10,11^, we provide strong evidence that CircS is not merely a cluster of comorbidities but also a distinct, independent predictor of respiratory decline. Our findings revealed that systemic circadian–metabolic disruption is longitudinally associated with the incidence of CLD, and the magnitude of this risk remains robust even after rigorously adjusting for traditional lifestyle confounders^20^. While the association is consistent across both cohorts, its phenotypic expression is context dependent; the effect was amplified in the British population, driven by a synergistic interaction with alcohol consumption and obesity, suggesting a significant interactive effect^21,22^.

Previous studies have examined primarily circadian markers (e.g., sleep duration and shift work) and metabolic dysfunction in isolation, often adopting an organ-centric perspective of respiratory health^23,24^. While the LUNG SAFE study and other studies have established the role of individual metabolic components in the decline in lung function, they failed to capture the cumulative physiological burden of desynchrony^25,26^. Our study advances this paradigm by confirming that CircS is a composite “sentinel indicator”^27^. Our findings are consistent with those of recent experimental models, which revealed that clock gene mutations compromise lung immunity, bridging the gap between molecular chronobiology and clinical epidemiology^28^.

While our observational data cannot establish causality, previous experimental models suggest that the association between CircS and incident CLD might involve dysregulation of the “clock–lung axis”. The molecular clockwork orchestrates important pulmonary defense mechanisms, including the resolution of airway inflammation, the secretion of mucus, and redox balance^3^.

First, circadian misalignment decreases the expression of nuclear factor erythroid 2-related factor 2 (Nrf2), the master regulator of antioxidant responses^3^. In the CircS state, the rhythmic activation of Nrf2 is weakened, increasing the susceptibility of lung tissue to oxidative stress caused by environmental factors^29^.

Second, systemic metabolic stress promotes chronic low-grade inflammation (“meta-inflammation”)^30^. The increases in visceral adiposity and insulin resistance drive the secretion of proinflammatory cytokines (IL-6 and TNF-α), which can enter the pulmonary circulation, priming the lungs for exaggerated inflammatory responses and tissue remodeling^31,32^. However, because these specific cardiometabolic traits are embedded within our composite CircS definition, our cohort data preclude formal mediation analyses to statistically isolate this pathway. Therefore, this meta-inflammation mechanism remains a plausible hypothesis in our study context.

A key finding of our study is the stronger effect size observed in the ELSA cohort than in the CHARLS cohort. This difference may be partially explained by the synergistic effect of clustered risk factors. The ELSA cohort exhibited a significantly higher baseline BMI and greater prevalence of alcohol consumption. Alcohol is a potent chronodisruptor that uncouples the central pacemaker in the suprachiasmatic nucleus from the peripheral clocks in the liver and lung^33,34^. When CircS compromises baseline circadian integrity, concurrent lifestyle stressors such as alcohol consumption or severe obesity may further overwhelm the residual compensatory mechanisms of the lungs^35,36^. This view is supported by our subgroup analysis involving the ELSA cohort, where the risk was disproportionately concentrated among individuals who consumed alcohol, suggesting that lifestyle stressors can synergistically amplify the pathogenicity of circadian misalignment.

The primary strength of this study is its dual-cohort design, wherein the findings are validated across East Asian and Western European populations, increasing the generalizability of our findings. The rigorous control for lifestyle factors and the application of sensitivity analyses with multiple imputation strengthened the robustness of our conclusions. Furthermore, the application of inverse probability of treatment weighting (IPTW) effectively addressed potential baseline imbalances, thereby strengthening the internal validity of the detected associations in our observational design. However, our study had several limitations. First, the diagnosis of CLD relied on self-reported physician diagnoses, which may have introduced recall bias, although this method has been validated in large epidemiological surveys^37^. Second, although we adjusted for smoking and indoor fuel use (in CHARLS), residual confounding effects from environmental pollutants cannot be ruled out. Specifically, while partial data on physical activity and detailed occupational or environmental exposures were collected in the original databases, the survey instruments and categorization criteria differed so fundamentally between CHARLS and ELSA that they could not be reliably harmonized for cross-cohort analysis. Additionally, there is inherent methodological heterogeneity in how central obesity and depression were defined across the two cohorts. Rather than compromising comparability, this adaptation was epidemiologically necessary. We applied ethnic-specific waist circumference thresholds on the basis of the International Diabetes Federation consensus, alongside culturally validated depression scales such as the CES-D 10 and CES-D 8. This strategy allowed us to capture the true biological and conceptual equivalence of the circadian–metabolic burden between distinct East Asian and Western European populations, whereas forcing identical raw metrics would have introduced severe misclassification bias.

Third, while we explicitly excluded participants with baseline CLD or asthma to minimize reverse causality, we cannot entirely rule out a bidirectional relationship between circadian disruption and lung health. Early subclinical respiratory symptoms such as nocturnal cough or mild dyspnea may disrupt sleep architecture, inadvertently fulfilling the criteria for CircS before a formal CLD diagnosis is made. Future studies utilizing continuous respiratory monitoring are needed to clarify this dynamic. Fourth, nonrespiratory mortality is a significant competing risk in these aging cohorts. Because exact mortality dates were frequently interval-censored or missing, we could not reliably perform competing risk regressions such as the Fine–Gray model. As a result, treating death as a standard censoring event in our Cox models may have slightly overestimated the absolute risk of incident CLD. Furthermore, the small number of participants with extreme CircS clustering (e.g., 6 or 7 components) prevented a reliable dose‒response analysis. Future large-scale studies are needed to determine whether respiratory risk increases continuously with increasing CircS severity. Finally, as this was an observational study, we can infer temporal precedence but cannot establish causal relationships^38,39^. Our analysis is fundamentally limited in its ability to elucidate the underlying biological mechanisms linking CircS to CLD incidence. The composite nature of CircS serves as a practical risk proxy rather than a specific mechanistic pathway. Future basic science and translational research are needed to investigate the molecular underpinnings of this association, including the exploratory alcohol interaction observed in our subgroups.

Clinically, these results complement the traditional isolation of lung health from systemic rhythmicity. Our findings showed that CircS is a reliable indicator of respiratory vulnerability. The presence of clustered circadian–metabolic disturbances should encourage clinicians to perform early respiratory screening, particularly in patients with coexisting obesity or those that consume alcohol. From a public health perspective, interventions must shift from simple smoking cessation to embracing a holistic chronomedicine approach. Strategies aiming to resynchronize the biological clock through optimized sleep hygiene^40^, time-restricted eating^41^, and the restriction of alcohol consumption to moderate levels may represent practical strategies to reduce the growing global burden of CLDs^42^.

## Conclusion

In summary, in this study, we found that CircS is a distinct and independent determinant of incident CLD in the aging population. The robustness of this association across genetically and culturally diverse cohorts highlights that systemic circadian dysregulation is a universal predictor of respiratory pathology. The synergistic interaction observed between circadian misalignment and alcohol consumption indicates an exploratory interactive effect, revealing that a specific subpopulation is at increased risk. From a clinical perspective, these findings broaden the perspective of preventive medicine, expanding the focus from organ-centric models to the recognition of circadian integrity as a critical target. Consequently, public health strategies that prioritize circadian alignment, specifically through the optimization of sleep hygiene, metabolic correction, and moderate alcohol consumption, are promising prophylactic approaches for mitigating the growing global burden of CLDs.

## Supplementary Information

Below is the link to the electronic supplementary material.

Supplementary Material 1

## Data Availability

The datasets generated and/or analyzed during the current study are available in public repositories. Data from the China Health and Retirement Longitudinal Study (CHARLS) are publicly available at http://charls.pku.edu.cn/en. Data from the English Longitudinal Study of Ageing (ELSA) are available through the UK Data Service at https://ukdataservice.ac.uk/ or the ELSA project website at https://www.elsa-project.ac.uk/. Access to these datasets requires registration with the respective repositories.

## Abbreviations

BMI: Body mass index
CES-D: Center for epidemiologic studies depression scale
CHARLS: China health and retirement longitudinal study
CI: Confidence interval
CircS: Circadian syndrome
CLD: Chronic lung disease
ELSA: English longitudinal study of ageing
HbA1c: Hemoglobin A1c (glycated hemoglobin)
HDL-C: High-density lipoprotein cholesterol
HR: Hazard ratio
IL-6: Interleukin-6
MAR: Missing at random
MICE: Multiple imputation by chained equations
Nrf2: Nuclear factor erythroid 2-related factor 2
SD: Standard deviation
TNF-α: Tumor necrosis factor-alpha
