# Supplementary material for: Prospective association between circadian syndrome and incident chronic lung disease in the CHARLS and ELSA cohorts
Source: Sci Rep. 2026 May 16;16:22304. doi: 10.1038/s41598-026-50994-1 (PMC13376350; doi:10.1038/s41598-026-50994-1)
Supplement: Supplementary file 1 — Supplementary Material 1 [file 41598_2026_50994_MOESM1_ESM.docx]

Table S1. Sensitivity analysis: Association between Circadian Syndrome (CircS) and incident CLD using Inverse Probability of Treatment Weighting (IPTW).

|  | CHARLS | | ELSA | |
| --- | --- | --- | --- | --- |
|  | *HR (95%CI)* | *P* | *HR (95%CI)* | *P* |
| Model 3 | 1.16(1.01-1.33) | 0.042 | 1.53(1.20-1.95) | <0.001 |
| IPTW Model | 1.18 (1.04-1.35) | 0.011 | 1.59 (1.24-2.05) | <0.001 |

**Model 3** (Fully Adjusted): Adjusted for sociodemographic factors (age, sex, marital status, and education level) and lifestyle factors (smoking status and alcohol consumption). **IPTW Model**: Utilized stabilized weights truncated at the 1st and 99th percentiles, with robust standard errors. Propensity scores for the IPTW model were estimated using age, sex, annual household expenditure, marital status, education level, smoking status, and alcohol consumption.
